# How Reliable Is the Alpha-defensin Immunoassay Test for Diagnosing Periprosthetic Joint Infection? A Prospective Study

**DOI:** 10.1007/s11999-016-4906-0

**Published:** 2016-06-24

**Authors:** Tommaso Bonanzinga, Akos Zahar, Michael Dütsch, Christian Lausmann, Daniel Kendoff, Thorsten Gehrke

**Affiliations:** 1 HELIOS ENDO Klinik, Holstenstrasse 2, 22767 Hamburg, Germany; 2 HELIOS Klinik Berlin-Buch, Berlin, Germany

## Abstract

**Background:**

A key issue in the treatment of periprosthetic joint infection (PJI) is the correct diagnosis. The main problem is lack of diagnostic tools able to diagnose a PJI with high accuracy. Alpha-defensin has been proposed as a possible solution, but in the current literature, there is a lack of independent validation.

**Questions/purposes:**

We performed a prospective study to determine (1) what is the sensitivity, specificity, and positive and the negative predictive values of the alpha-defensin immunoassay test in diagnosing PJI; and (2) which clinical features may be responsible for false-positive and false-negative results?

**Methods:**

Preoperative aspiration was performed in all patients presenting with a painful hip/knee arthroplasty, including both primary and revision implants. Metallosis, other inflammatory comorbidities, and previous/concomitant antibiotic therapy were not considered as exclusion criteria. An inadequate amount of synovial fluid for culture was an exclusion criterion. A total of 156 patients (65 knees, 91 hips) were included in this prospective study. At the time of revision, synovial fluid samples were taken to perform the alpha-defensin assay. During surgical débridement of tissue, samples for cultures and histologic evaluation were taken, and samples were cultured until positive or until negative at 14 days. A diagnosis of PJI was confirmed in 29 patients according to the International Consensus Group on PJI.

**Results:**

The sensitivity of the alpha-defensin immunoassay was 97% (95% confidence interval [CI], 92%–99%), the specificity was 97% (95% CI, 92%–99%), the positive predictive value was 88% (95% CI, 81%–92%), and the negative predictive value was 99% (95% CI, 96%–99%). Among four false-positive patients, two had metallosis and one had polyethylene wear. The false-negative case presented with a draining sinus, and intraoperative cultures were also negative.

**Conclusions:**

Alpha-defensin assay appears to be a reliable test, but followup evaluation is needed to estimate longer term performance of the test. The authors believe that alpha-defensin has demonstrated itself to be sufficiently robust that PJI diagnostic criteria now should include this test. Future studies are needed to compare the differences among the diagnostic capability of the available tests, in particular when metallosis is present, because metallosis may predispose the test to a false-positive result.

**Level of Evidence:**

Level I, diagnostic study.

## Introduction

Periprosthetic joint infection (PJI) is one of the biggest challenges in orthopaedic surgery today. PJI is reported to be the cause of failure for 25% [4] of TKA and 15% [3] of THA. Given the expected increasing incidence and the economic impact of PJI [18], a strong effort has been recently made by the international orthopaedic community to improve the management of this complication of arthroplasty [5, 20]. A key issue in the treatment of PJI is making the correct diagnosis as early as possible. Because the most common symptom of PJI is nonspecific pain, many tests are used today in an attempt to find the cause of pain and to differentiate between septic and aseptic revision surgery with various results [12]. Historically, direct tissue cultures have been considered the diagnostic standard; however, these require surgery to be collected and are neither completely sensitive nor perfectly specific [23]. In an attempt to guide clinicians in everyday practice, the Musculoskeletal Infection Society (MSIS) has published a new diagnostic approach with two existing major or six minor criteria for diagnosis of PJI [20]. This definition of PJI was recently revised by the International Consensus Group on Periprosthetic Joint Infection (Table 1) [5]. According to the PJI Consensus Group, patients should be considered to have PJI if they meet one of the major criteria or at least three of the minor criteria [5] (Table 2).

**Table 1:** Definition of periprosthetic joint infection (PJI) according to the International Consensus Group

PJI is present when one of the major criteria exists or three of five minor criteria exist	
Major criteria	Two positive periprosthetic cultures with phenotypically identical organisms, OR A sinus tract communicating with the joint, OR
Minor criteria	(1) Elevated serum C-reactive protein AND erythrocyte sedimentation rate (2) Elevated synovial fluid white blood cell (WBC) count OR ++change on leukocyte esterase test strip (3) Elevated synovial fluid polymorphonuclear neutrophil percentage (4) Positive histological analysis of periprosthetic tissue (5) A single positive culture

Reprinted from The Journal of Arthroplasty, 29(7), Parvizi J, Gehrke T, Definition of periprosthetic joint infection, Page 1331, Copyright 2014, with permission from Elsevier.

**Table 2:** The threshold for the minor diagnostic criteria according to the International Consensus Group

Criterion	Acute PJI (< 90 days)	Chronic PJI (> 90 days)
Erythrocyte sedimentation rate (mm/hr)	Not helpful; no threshold was determined	30
C-reactive protein (mg/L)	100	10
Synovia white blood cell count (cells/μL)	10,000	3,000
Synovial polymorphonuclear percentage (%)	90	80
Leukocyte esterase	+ or ++	+ or ++
Histological analysis of tissue	> 5 neutrophils per high-power field in 5 high-power fields (×400)	Same as acute

Reprinted from The Journal of Arthroplasty, 29(7), Parvizi J, Gehrke T, Definition of periprosthetic joint infection, Page 1331, Copyright 2014, with permission from Elsevier; PJI = periprosthetic joint infection.

Recently, the diagnostic capability of synovial fluid biomarkers has been highlighted as a possible breakthrough in this scenario. Promising results have been reported about alpha-defensin, which is protein naturally released by neutrophils in response to synovial fluid pathogens [14]. The sensitivity and the specificity of the alpha-defensin immunoassay test have been reported to be above 96% [7–10]. Furthermore, it has been demonstrated that a wide spectrum of organisms triggers the level of alpha-defensin in the synovial fluid [11]. However, to the best of our knowledge, with only a couple of exceptions [2, 13], the research on the alpha-defensin immunoassay test for PJI diagnosis has been published exclusively by its developers. Because the sample size of those two studies was quite limited, there is still lack of independent validation.

Therefore, we performed a prospective study to answer the following questions: (1) What is the sensitivity, specificity, positive predictive value, and negative predictive value of the alpha-defensin immunoassay test in diagnosing a PJI; and (2) which clinical features may be responsible for false-positive and false-negative alpha-defensin assay results?

## Patients and Methods

After approval of the local ethical committee, a prospective analysis of data collected from the ENDO Klinik, Hamburg, Germany, was performed in all the patients with a chronically (> 90 days) [5] painful knee or hip arthroplasty who underwent revision surgery from April to October 2015. Patients with primary arthroplasties and revision arthroplasties both were considered for inclusion. No acute revisions (symptoms of less than 90 days’ duration) [5] were enrolled into the study, because to our knowledge, alpha-defensin cutoff levels for acute or early postoperative infection were not known when we began this study. Informed consent was obtained for each patient enrolled in the study.

Patients’ histories, clinical evaluations, laboratory examinations including C-reactive protein (CRP), and joint aspiration fluid were collected preoperatively as routine diagnostic procedures. Metallosis, other inflammatory comorbidities, and previous or concomitant antibiotic therapy were not considered as exclusion criteria. An inadequate amount of synovial fluid for culture was considered an exclusion criterion. If the amount of fluid was enough, synovial fluid cell count including granulocyte percentage and leukocyte esterase (LE) test was performed as well. In selected patients in whom clinical evaluation and other minor criteria [5] (Table 2) suggested the presence of infection, but who had negative fluid cultures, preoperative tissue biopsies were taken through an open surgical procedure before the planned one-stage revision. At the time of revision procedure, standardized synovial fluid samples were taken to perform the alpha-defensin assay test. If no fluid could be obtained by intraoperative aspiration, the patient was excluded from the study. After the surgical procedure and the intraoperative aspiration, 156 patients (90 females, 66 males) presenting with 156 painful total joint arthroplasties (65 TKAs, 91 THAs) were included in the study.

Every patient underwent the standard preoperative diagnostic protocol of ENDO Klinik [15] with blood tests for CRP and joint aspiration of the painful joint. From the aspirate standard microbiology cultures, cell count and granulocyte percentage evaluation were performed. Before the aspiration, patients were not allowed to take antibiotics for 2 weeks (antibiotic holiday) and the bacterial samples were cultured for 14 days in the Microbiology Laboratory of University Hospital Schleswig-Holstein, Kiel, Germany. Based on the findings of the preoperative diagnostic tests, the patients were discussed in the institutional multidisciplinary team meeting and arthroplasties were considered as aseptic or septic according to the diagnostic criteria of the modified PJI Consensus Group diagnostic protocol [5]. Members of the multidisciplinary team were orthopaedic surgeons and a microbiology and infectious disease consultant. If a patient had a positive culture, but all other parameters were negative, the culture result was considered a false-positive (contaminant), and the revision was planned as aseptic.

**Fig. 1:**
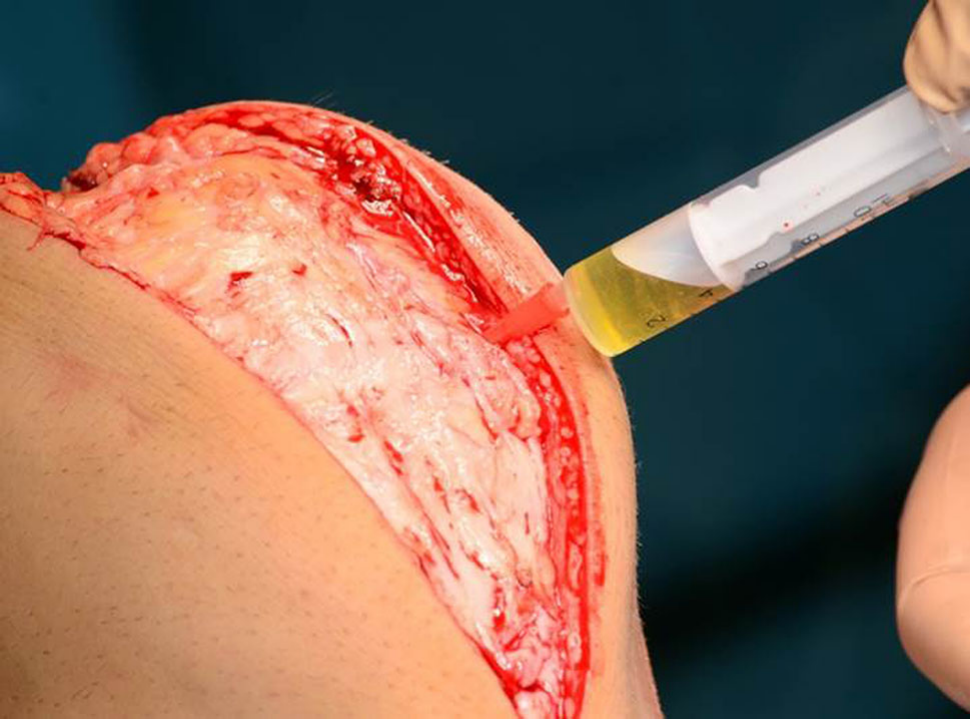
Intraoperative aspiration was performed through the capsule right before capsulotomy, avoiding admixture of blood. The knee is shown after soft tissue dissection.

After admission to the ENDO Klinik, all study patients underwent revision surgery. Patients who were considered free from PJI based on the criteria evaluable on preoperative aspiration (Table 2) and clinical evaluation were revised as though there was no infection present with partial or total exchange of the failed components as indicated (cup and/or stem exchange after THA or femoral and/or tibial exchange after TKA). Patients diagnosed as having PJI underwent a single-stage direct exchange following the ENDO Klinik protocol for PJI if microorganisms were known in advance or in a two-stage revision if the bacteriology was not known after preoperative diagnostics [15]. One of the authors (MD) collected all the aspirates and patient data as part of this prospective study design. The intraoperative aspiration was performed after surgical incision, preparation of soft tissues, and exposure of the capsule without opening the joint (Fig. 1). A 20-mL syringe and an 18-gauge needle were used to carefully obtain the synovial fluid avoiding an admixture of blood. The synovial fluid samples were sent to an independent blinded laboratory (Labor Dr. Fenner und Kollegen, Hamburg, Germany) within 24 hours [9]. The test kits were provided to the independent laboratory free of charge by CD Diagnostics (Claymont, DE, USA). During the surgical débridement in both septic and aseptic revisions, at least three tissue samples for cultures were taken from different regions of the surgical field according to a previously described protocol [15]. In the group of patients suspected as having PJI based on the preoperative multidisciplinary team meeting, at least two samples for histological examination were taken as well. Patients were considered as potentially having a PJI when one of the major criteria or four of the minor criteria based on the diagnostic criteria of the PJI Consensus Group [5] are given. This preoperative suspicion was then proven by intraoperative findings. After 14 days, the results of the intraoperative cultures and histological findings were collected and these records were added to the database. Both the microbiologist and the pathologist were blinded to each patient’s clinical status and to the alpha-defensin level. The pathologist was not informed about the alpha-defensin level and the bacterial culture before final histology finding. The microbiologist was not informed about the histology finding. Metallosis, defined as an intraoperative macroscopic finding with gray- or black-stained synovial tissue, which was confirmed by histological investigation after tissue sampling, was not considered as an exclusion criterion.

**Fig. 2:**
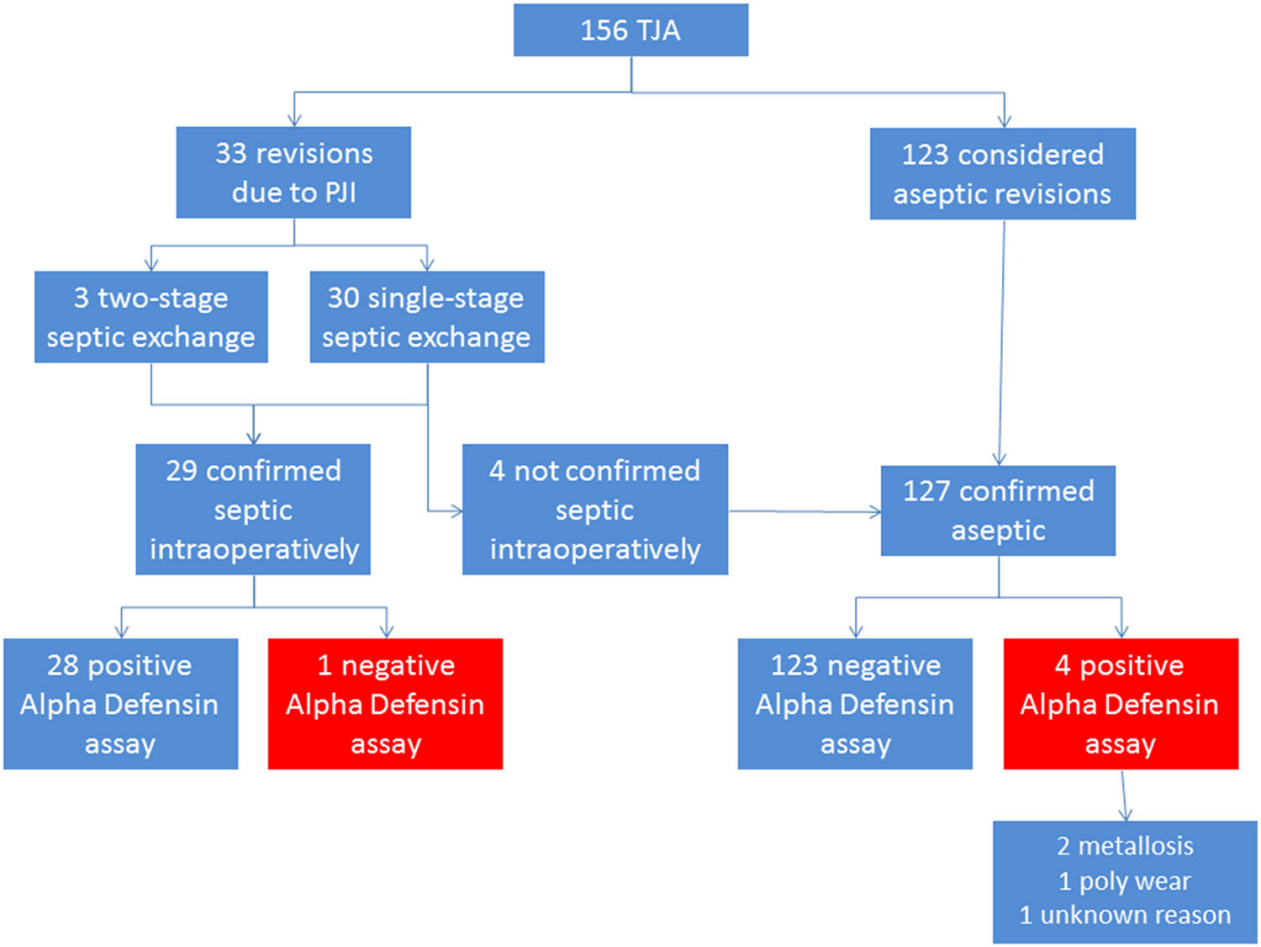
Workflow describing the features of the patients included in the study. Reproduced with permission from Silvia Bassini.

A preoperative biopsy was performed in four patients to find the causative organism of the suspected PJI. After preoperative evaluation by the multidisciplinary team, 33 patients were considered to have a PJI. Thirty patients (91%) underwent a one-stage direct exchange for infection; in three patients, the first stage of a two-stage revision (9%) was performed: the implants were removed, thorough débridement was performed, and a customized antibiotic spacer was inserted. Seven patients (21%) presented with a draining sinus. The diagnosis of PJI was confirmed by intraoperative microbiology and histology findings in a total of 29 patients (Fig. 2). Several microorganisms were isolated from preoperative and intraoperative cultures (Table 3). Twenty-seven of them matched the major criteria for PJI according to the PJI Consensus Group protocol. In two cases the intraoperative culture failed to show growth and no draining sinus was present; however, the diagnosis was confirmed by preoperative positive culture, elevated CRP, and intraoperative positive histology, matching the PJI Consensus Group minor criteria. In both cases, a preoperative antibiotic therapy was started as a result of a high risk of septicemia. Among these 29 patients, the CRP was negative in five (17%) patients.

**Table 3:** Microorganisms isolated from patients with PJI

Microorganism	Number	Percentage
*Staphylococcus epidermidis*	12	41
*Enterococcus faecalis*	4	14
*Staphylococcus hominis*	3	10
*Streptococcus agalactiae*	3	10
*Pseudomonas aeruginosa*	2	7
*Staphylococcus aureus*	2	7
*Streptococcus dysgalactiae*	2	7
*Staphylococcus capitis*	2	7
*Staphylococcus caprae*	2	7
*Propionibacterium avidum*	2	7
*Proteus mirabilis*	1	3
*Staphylococcus lugdunensis*	1	3
*Stenotrophomonas maltophilia*	1	3

PJI = periprosthetic joint infection.

The remaining 123 patients were diagnosed as not having PJI and underwent revisions for other indications (mostly implant loosening). In the series of patients free form PJI, the CRP was elevated in 10 cases (8%). Coexisting metallosis was found in 13 patients (11 without apparent infection, two apparently with infection). The diagnosis of metallosis was proven by intraoperative observation by the surgeon and histological study of tissue samples. Systemic inflammatory comorbidities were recorded as well (Table 4).

**Table 4:** Inflammatory disease as a comorbidity factor in the prospective series of 156 patients*

Patient number	Comorbitity	Revision	CRP (mg/L)	αD (S/CO)
1	Atopic eczema	Aseptic	0.94	0.2
2	Irregular antibodies	Aseptic	1.04	< 0.1
3	Crohn’s disease	Aseptic	0.59	< 0.1
4	Rheumatoid arthritis	PJI	26.5	7.1
5	Chronic lymphatic leukemia	Aseptic	3.1	< 0.1
6	Psoriasis	Aseptic	9.77	< 0.1
7	Psoriasis	Aseptic	5.88	< 0.1
8	Rheumatoid arthritis	Aseptic	1.67	< 0.1
9	Lupus erythematodes	Aseptic	3.03	< 0.1

*Nine patients had a relevant systemic disease; alpha-defensin (αD) was only elevated in one patient with proven PJI; CRP = C-reactive protein; PJI = periprosthetic joint infection.

### Statistical Analysis

The results of the alpha-defensin assay were reported as a semiquantitative signal-to-cutoff ratio of 1.0 as a threshold for PJI diagnosis. To statistically assess the performance of the current test, the specificity, sensitivity, positive predictive value, and negative predictive value were evaluated.

In particular, specificity indicates the percentage of subjects without the disease who get a negative test result; sensitivity indicates the percentage of subjects with the disease who get a positive test result; positive predictive value is the probability that the disease is present in case of a positive test; negative predictive value is the probability that the disease is not present in case of a negative test. The 95% confidence interval (95% CI) has been calculated for each of the previous statistical measures.

## Results

**Fig. 3:**
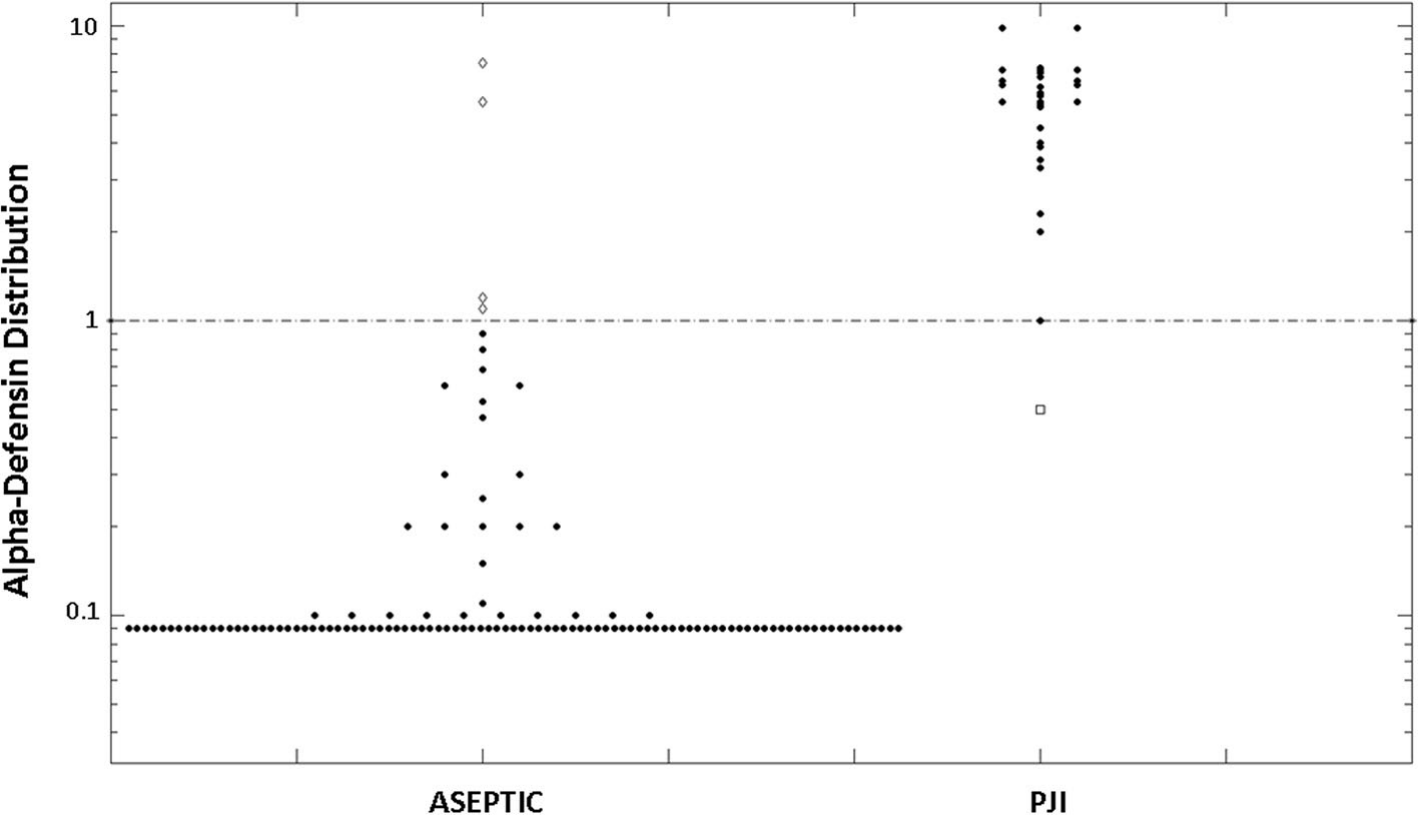
Synovial fluid alpha-defensin values (logarithmic scale) for aseptic and PJI patients are shown separately. The line indicates the alpha-defensin diagnostic threshold of 1.0 (signal-to-cutoff ratio [S/CO]). The five white dots represent the misdiagnosed patients, being false-negative (in the PJI group) or false-positive (in the aseptic group).

The alpha-defensin assay was positive in 32 joints and negative in 124 (Fig. 2). When matching these data with the diagnosis based on the PJI Consensus Group criteria, it resulted in the alpha-defensin assay being false-positive in four cases and false-negative in only one case (Fig. 3). Statistical analysis revealed that the sensitivity of the alpha-defensin immunoassay was 97% (95% CI, 92%–99%), the specificity was 97% (95% CI, 92%–99%), the positive predictive value was 88% (95% CI, 81%–92%), and the negative predictive value was 99% (95% CI, 96%–99%).

**Fig. 4:**
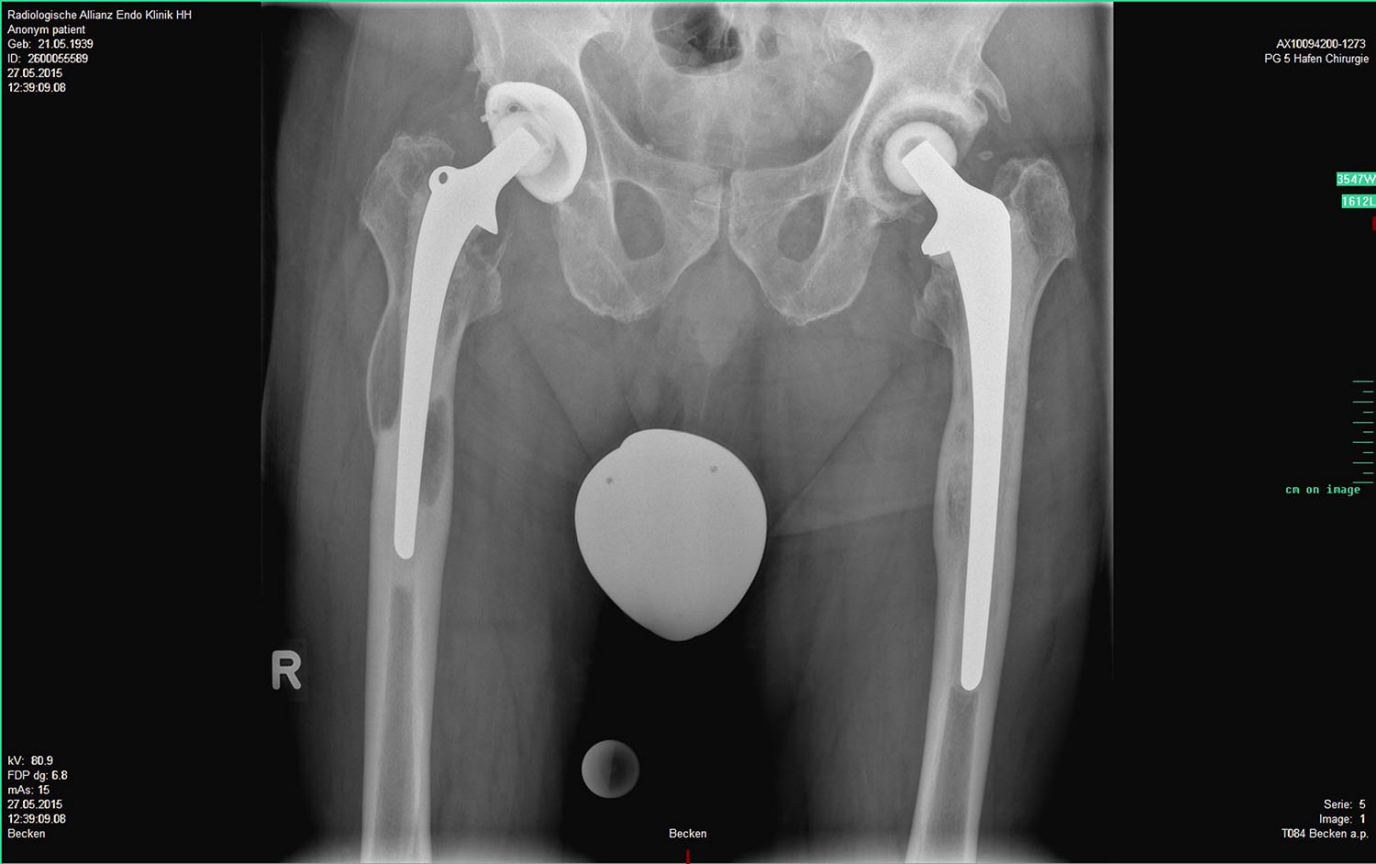
Preoperative AP radiograph of a patient with false-positive alpha-defensin value reveals polyethylene wear and osteolysis of the proximal femur at his right THA.

Among the four patients with a false-positive alpha-defensin assay, two had a coexisting metallosis and one had severe polyethylene wear with osteolysis (Fig. 4). In one patient no particular clinical feature was noticed. The two cases with metallosis had a negative CRP, whereas the patient with polyethylene wear had a CRP of 15 mg/L. Cell count and LE tests were not available for any of these three patients. The false-negative case presented with a draining sinus; the intraoperative cultures were negative but the CRP was 15 mg/L and the granulocyte percentage was 80%.

## Discussion

Although several strategies are available today for surgical management of PJI, the diagnosis remains challenging as a result of the lack of diagnostic tools able to diagnose PJI with reliable specificity and sensitivity [20]. Recently, synovial fluid biomarkers such as alpha-defensin have been proposed as a possible solution in this complex scenario with very promising results [8–11]. In the current series, the alpha-defensin immunoassay has shown both sensitivity and specificity as high as 97%. Furthermore, the positive and negative predictive values were, respectively, 88% and 99%, demonstrating outstanding performance of alpha-defensin if the result is negative; in other words, if the alpha-defensin test is negative, it is quite likely that the pain in the joint after THA or TKA is not caused by PJI. If the result is positive, the likelihood of PJI is very high, but other reasons for elevated alpha-defensin level should be considered and excluded.

The authors noted some limitations of the current study. First, the sample size was quite small compared with other studies on PJI diagnosis [1, 16, 21]. However, when looking at the available research, this was the largest single-center study evaluating alpha-defensin assay. Furthermore, this was the only single-center series of which we are aware that included patients with metallosis, wear, and inflammatory comorbidities. Second, all clinical data exploitable for the PJI Consensus Group criteria were not available for all patients, but this is a common problem in clinical practice because the aspiration fluid is often not adequate in quantity and/or quality to perform all required tests. The lack of some tests combined with the size of the series did not allow for statistical comparison of diagnostic capability between the alpha-defensin assay and other tests. However, for all patients who received a positive alpha-defensin test, there was a sufficient amount of data to confirm or rule out the infection according to the PJI Consensus Group criteria. Third, the synovial fluid samples used to perform the alpha-defensin assay test were collected intraoperatively after surgical incision and dissection. This was an ideal condition in which the aspiration was performed directly through the capsule but not reproducible in everyday clinical practice, like in the outpatient clinic. Fourth, erythrocyte sedimentation rate (ESR) was not available for any patient because in our institution, it is not routinely performed in patients undergoing workup for PJI. However, it has been proved that ESR is not specific for PJI with a reported specificity of 68% to 87% [1, 6, 16, 17, 19, 22]. Finally, our results should be considered a best-case estimate of the test’s performance, because there was no extended surveillance here, and so it remains possible that some of the patients diagnosed as being without PJI may indeed have an indolent infection and present later. Future studies with longer followup clearly are called for to address this issue.

The results of the current article are consistent with the limited available research on this topic. First, Deirmengian et al. [10] have tested 16 possible synovial biomarkers of 95 patients (66 patients with aseptic complications, 29 patients with PJI) and reported that five biomarkers, including alpha-defensin, provided a diagnosis that matched with the MSIS criteria for the whole series. In this study, patients presenting with adverse reactions to metal debris were excluded, whereas no inflammatory comorbidity was considered as exclusion criteria. Bingham et al. [2] have reported even better results for the alpha-defensin assay alone in 61 aspirations (19 septic, 42 aseptic aspirates). In their series, the sensitivity was 100% (95% CI, 79%–100%) and the specificity was 95% (95% CI, 83%–99%). Patients with a concomitant autoimmune disease were excluded and other comorbidities were not mentioned. However, they failed to show any significant difference in ruling out PJI except with respect to ESR; this latter test was not performed in the current study. More recently, Frangiamore et al. [13] reported about a mixed series combining single-stage revisions with reimplantations at two-stage revision. The sensitivity and the specificity for the single-stage group were 100% (95% CI, 86%–100%) and 95% (95% CI, 90%–100%), respectively. Interestingly, they noticed that the performance of the alpha-defensin assay was poorer in the second-stage group, especially concerning the sensitivity that went down to 67% (95% CI, 12%–95%). However, the authors noticed the sample size was too small to allow for appropriate evaluation. Another report by Deirmengian et al. [9], including patients with coexisting metallosis and inflammatory comorbidities, gained results similar to the current paper. In a series of 149 patients (112 patients with an aseptic complication, 37 patients with PJI), the specificity and the sensitivity were reported to be 96% (95% CI, 90%–99%) and 97% (95% CI, 86%–99.6%), respectively. Interestingly, it was noted that including the value of synovial fluid CRP in diagnostic algorithm, the overall specificity increased to 100% (95% CI, 96.7%–100%).

Another finding of the current study is that among the four reported false-positive arthroplasties, two patients had a coexisting metallosis, but alpha-defensin was not influenced by any systemic inflammatory disease (Table 4). Deirmengian et al. [9] reported on three hips revised for metallosis out of five false-positive joints. These data highlight that metallosis could be a misleading factor in reading alpha-defensin assay results. However, the alpha-defensin value was not elevated in most of the patients diagnosed as being without infection with a coexisting metallosis in either series. The other two papers reporting misclassified patients did exclude patients with a metallosis. Bingham et al. [2] recorded two patients with false-positive results. In those patients, other markers of inflammation such as CRP, cell count, and ESR were elevated as well. They theorized that aseptic inflammation might be responsible for elevated alpha-defensin levels. Frangiamore et al. [13] observed two false-positive results; however, one patient was undergoing a second-stage revision and as the authors recognized, this biomarker assay is designed only for the first operation of a two-stage exchange for infection or a single-stage revision. A possible interpretation of these data could be that in case of elevated synovial alpha-defensin levels and less than three minor criteria according to the PJI Consensus Group, the presence of possible metal debris should be considered.

Like in the current series, Deirmengian et al. [9] also reported only one false-negative. In both series, the cultures were negative but the diagnosis was done by matching other PJI Consensus Group criteria. In the series of Frangiamore et al. [13], there was one false-negative patient as well; this subject had a positive culture but it was considered contamination.

In conclusion, the findings of this study suggest that the alpha-defensin assay has a role to play in the complex scenario of PJI diagnosis. However, like with all the other available tests, it is not the perfect diagnostic tool with 100% specificity and sensitivity, but this independent confirmation of the performance of alpha-defensin suggests that it could be integrated among the existing PJI diagnostic criteria. However, our results should be considered a best-case estimate of the test’s performance, because there was no followup evaluation. Therefore, it remains possible that some of the patients diagnosed as being without PJI may indeed have a chronic, indolent infection and present later. Future studies with larger series are needed to statistically compare the difference among the diagnostic capabilities of the available tests. Particular effort is needed for those patients presenting with a coexisting metallosis, which may predispose the test to a false-positive result.
